# Supplementary figures and images for: A cross-sectional study of social inequities in medical crowdfunding campaigns in the United States
Source: PLoS One. 2020 Mar 5;15(3):e0229760. doi: 10.1371/journal.pone.0229760 (PMC7058302; doi:10.1371/journal.pone.0229760)

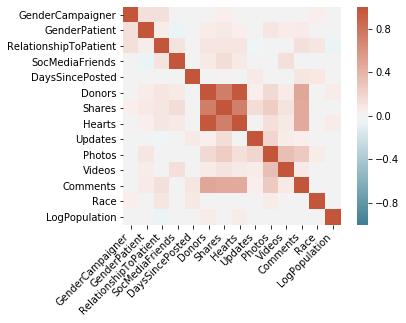

Supplement: S1 Fig — Based on Spearman’s ranked correlation test used to test the independence of each campaign feature used in tests. (PNG) [file pone.0229760.s001.png]
